# Availability and trend of dissemination of cardiac rehabilitation in China: report from the multicenter national registration platform between 2012 and 2021

**DOI:** 10.3389/fcvm.2023.1210068

**Published:** 2023-06-19

**Authors:** Sisi Zhang, Rongjing Ding, Sikun Chen, Xiaoping Meng, Li Jianchao, Dao Wen Wang, Dayi Hu

**Affiliations:** ^1^ Division of Cardiology, Department of Internal Medicine, Tongji Hospital, Tongji Medical College, Huazhong University of Science and Technology, Wuhan, China; ^2^ Department of Rehabilitation, Peking Union Medical College Hospital, Chinese Academy of Medical Sciences, Beijing, China; ^3^ Institute of Clinical Epidemiology, Key Laboratory of Public Health Safety, Ministry of Education, Key Lab of Health Technology Assessment of Ministry of Health, School of Public Health, Fudan University, Shanghai, China; ^4^ Department of Cardiology and Cardiac Rehabilitation, The Affiliated Hospital of Traditional Chinese Medicine, Changchun, China; ^5^ School of Engineering Medicine, Beijing Advanced Innovation for Biomedical Engineering, Beihang University, Beijing, China

**Keywords:** cardiac rehabilitation, coronary heart disease, exercise capacity, density, availability

## Abstract

The study aimed to evaluate the current status of cardiac rehabilitation programs in China by registering and tracking patients undergoing CR programs in the database. Data were extracted from the online registry platform of the China Society of Cardiopulmonary Prevention and Rehabilitation from February 2012 to December 2021. Overall, data on 19,896 patients with cardiovascular diseases (CVDs) from 159 hospitals in 34 provinces of China were extracted. From a time point of view, the number of patients who had undergone CR and institutions that perform CR showed the first decline in 2009 and then increased until 2021. From a geographic point of view, the degree of participation varied greatly among regions, most of which were concentrated in eastern parts of China. A higher population of patients who underwent CR were male, aged less than 60 years, with low-a risk for coronary heart disease (CHD), and tended to choose the hospital-based CR program among all cases registered in the database. The top three diseases in the patients who participated in CR were CHD, hypertension, and metabolic syndrome (MS). Centers with CR were more likely to be tertiary-level hospitals. After adjusting for baseline values, there were significant differences in post-CR exercise capacity among the three groups (home-based CR group, hospital-based CR group, and hybrid CR group), which were in favor of the hybrid CR group compared with other groups. The underutilization of CR is a global issue, not just in China. Despite the number of CR programs showing an increasing trend in the past years, CR in China is still in the preliminary stage of development. Furthermore, the participation of CR in China shows wide diversity across geography, disease, age, sex, risk stratification, and hospital-level factors. These findings reinforce the importance of the implementation of effective measures to improve the participation, enrollment in, and uptake of cardiac rehabilitation.

## Introduction

Cardiovascular diseases (CVDs) have become the leading cause of death worldwide, accounting for 40% of the deaths in the Chinese population (1, 2). According to the latest report of the National Center for Cardiovascular Diseases, there are 330 million patients with CVDs in China (3). With the rapid evolution of revascularization and the application of effective and advanced pharmacotherapy, more patients survive acute CVD events and live with this condition chronically. Therefore, there is a great need to build comprehensive, multidisciplinary prevention approaches to further stem this growing epidemic and reduce the occurrence and recurrence rate of CVD events.

Cardiac rehabilitation (CR) is a cost-effective secondary prevention model that delivers risk factor management strategies, psychosocial counseling, behavior modification, patient education, and supervised exercise, showing 20% reductions in cardiovascular mortality and morbidity (4–6). Multinational guidelines recommend CR in clinical practice (7–10). Despite these guidelines and well-established benefits, the availability of CR remains low, carried out only in 54.7% of institutions worldwide and 22.1% in low- and middle-income countries (LMICs), which have the greatest death rate from CVDs (11, 12). As reported, the enrollment rate differs greatly between Asian population and Western countries (13). In Singapore, only 12.3% of eligible patients participate in CR, while 14%‒50% participate in Korea and Japan (14, 15). The participants are also in suboptimal conditions with significant geographical variations and diversity of diseases. China is the world’s most populous developing country, accounting for approximately one-fifth of the total world population. In China, only 24% of hospitals had operational CR programs in 2016, and 216 medical institutions were developing CR programs in 2008, which is still significantly lower than that in most Western countries (e.g., the United States, Canada, South America, and the United Kingdom) (16).

In the past 5 years, under the leadership of the Committee of Cardiac Rehabilitation and Prevention of the Chinese Association of Rehabilitation Medicine, more than 500 CR centers have been established with unsurpassed speed globally (17). Therefore, quality monitoring and improvement are urgently needed.

To date, only few studies have ascertained the availability and state of CR at the national level in China. To improve the quality of secondary prevention and rehabilitation for cardiovascular diseases, the committee established a registry system for CR in 2012. This study aimed to report the implementation, availability, distribution (across different provinces, prefectures, and counties), enrollment, and trend of dissemination of the CR program among Chinese patients and further clarify the current state of CR in China by registering and tracking patients undergoing CR programs in the database.

## Materials and methods

### Data source and collection

We analyzed data from the China Society of Cardiopulmonary Prevention and Rehabilitation (CACPR) Registry Platform (http://www.cacpr.cn).

This nationwide registry platform was initiated in 2012 by the cardiopulmonary rehabilitation group of the Chinese Association of Rehabilitation Medicine. As a nationally representative database, CACPR was designed to monitor and improve the quality of CR throughout China, further reducing mortality and morbidity in patients with chronic cardiac diseases.

All members of the Committee of Cardiac Rehabilitation and Prevention of the Chinese Association of Rehabilitation Medicine are responsible for implementing and using this database and registering all patients in their affiliated institutions undergoing CR programs for CVD as the requirements to maintain member status. Following a diagnosis of CVD and CR assessment, professionals and/or clinicians who completed a short training course could enter their clinical data manually into the registry platform.

Data are reported on patients, institutions, and regional levels, and performed up to standards to support studies within the field of CR. We recorded patient demographic values, including age, sex, diagnoses with the International Classification of Diseases, 10th edition code (stable angina, prior myocardial infarction (MI), prior percutaneous coronary intervention, prior coronary artery bypass graft surgery), general comorbidities (such as hypertension, diabetes, obstructive sleep apnea syndrome, hypercholesteremia, and metabolic syndrome), baseline levels of clinical measures (including height, weight, body mass index, waist circumference, systolic blood pressure, diastolic blood pressure, total cholesterol, triglycerides, low-density lipoprotein, high-density lipoprotein, hemoglobin A1c, alanine aminotransferase, creatine kinase, fasting blood glucose), and medication usage (for example, the use of aspirin, beta-blockers, statin, nitrates, and adenosine-diphosphate receptor antagonists). In addition, we collected functional exercise capacity as measured by 6 min walking test (6 MWT), peak oxygen consumption (peak VO_2_), and oxygen consumption at the anaerobic threshold (VO_2_@AT) from cardiopulmonary exercise testing (CPET). We also recorded the date of CR enrollment, completion of follow-up, and the total number of follow-up visits.

Institutions certified for CR by the Chinese Association of Rehabilitation Medicine are stratified according to size, level (secondary, tertiary, and primary care facilities), and geographic region. Based on sociodemographic status and economic conditions, the regions from 34 provinces and administrative units of China (23 provinces, four municipalities, five autonomous regions, and two special administrative regions (SARs)) were divided into eastern, central, western, and Hong Kong, Macao, and Taiwan as the National Development and Reform Commission.

Exercise capacity data were measured at baseline, completion, and follow-up to monitor CR progression and outcome.

All data were uploaded according to the standard process and collected annually. The committee secretariat will regularly announce registered numbers at conferences to encourage participating institutions to input many more eligible cases.

Each institution pooled its own data to the server, while the system manager in the central database had access to retrieve all the data from the server through the web system in Microsoft Excel format and analyzed it. The system is equipped with an automatic identification function for outliers. The data were collected from February 2012 to December 2021. The requirement for written informed consent was waived.

### Study population

The subjects will be required to meet the following inclusion criteria: (1) age ≥18 years; (2) registered in the CACPR between 2012 and 2021 with available data; (3) admitted to the hospital with a diagnosis of coronary heart disease (CHD) (including unstable angina, ST-elevation MI (STEMI), or non-ST-elevation MI (NSTEMI) treated with an invasive procedure, cardiac surgery, or medicine alone), which followed the latest guidelines in use at that time. All patients were stratified as low-, middle-, and high-risk according to the risk algorithm developed by the American Association of Cardiovascular and Pulmonary Rehabilitation (18). Patients who died during hospitalization and patients who dropped out of the CR will be excluded from the study.

### Cardiac rehabilitation

The CR models include traditional hospital-based CR, home-based CR, and hyrid programs. Combining center-based and home-based settings can be called “hybrid rehabilitation”, which is effective in controlling cardiac risk factors.

All CR programs are recommended by the Chinese expert’s consensus on cardiac rehabilitation/secondary prevention for coronary artery disease, including psychological interventions, prescribed exercise training, nutrition, smoking cessation, and adherence to medications (19). All CHD patients followed the aerobic and resistance exercise training 3 times a week for 12 weeks. Each training session will consist of 5–10 min of warm-up, 20–60 min of aerobic and 10 min of resistance training, and 5–10 min of cool down. The intensity of aerobic exercise was close to an intensity of 50%−75% peak VO_2_. Resistance training should be moderate intensity based on the Borg Rating of Perceived Exertion (BORG) scale (20).

### Follow-up

Follow-up was performed after the patient completed three months of CR.

### Statistical analyses

Descriptive statistics (counts, percentages, or quartiles) were used to characterize CR availability, trends, and densities at different levels. We calculated the proportion of CR with regard to time, location (province, region, and hospital), population (age, sex, main disease, and risk stratification), and type of CR. Continuous variables are expressed as mean ± standard deviation (SD) and categorical data as percentages. Patients with missing data were excluded. Analysis of covariance (ANCOVA) was performed using the baseline values as covariates to compare the differences between groups (home-based CR, hospital-based CR, and hybrid CR) regarding exercise capacity (6MWT and peak VO_2_). The SPSS 26.0 (SPSS Inc., Chicago, IL) and GraphPad Prism 8.0 software were used for statistical analyses and plotting of graphs, respectively. *P *< 0.05 was considered statistically significant.

## Results

### Temporal distribution

**Figure 1:**
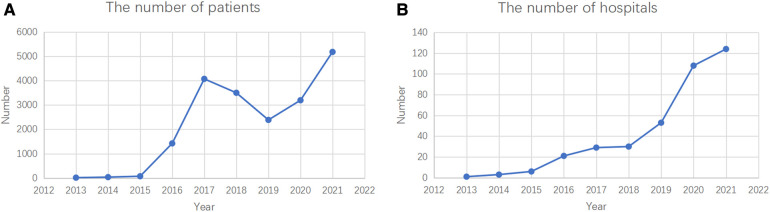
Dynamic trends in patients (A) and institutions (B) which performed CR in CACPR by National and Regional Levels, 2012–2021.

Figure 1 shows the time series of the CR population and institutions from 2012 to 2021. The annual number of patients increased from 15 in 2013 to 5,183 in 2021, while the number of institutions increased from 1 in 2013 to 149 in 2021. During the study period, the overall CR followed a growth trend, except for 2019. From 2015 to 2018, the CR proportion in China showed an upward trend. Subsequently, a short-term decline occurred in 2019, after which the number began to rise again until 2021.

### Regional distribution

**Figure 2:**
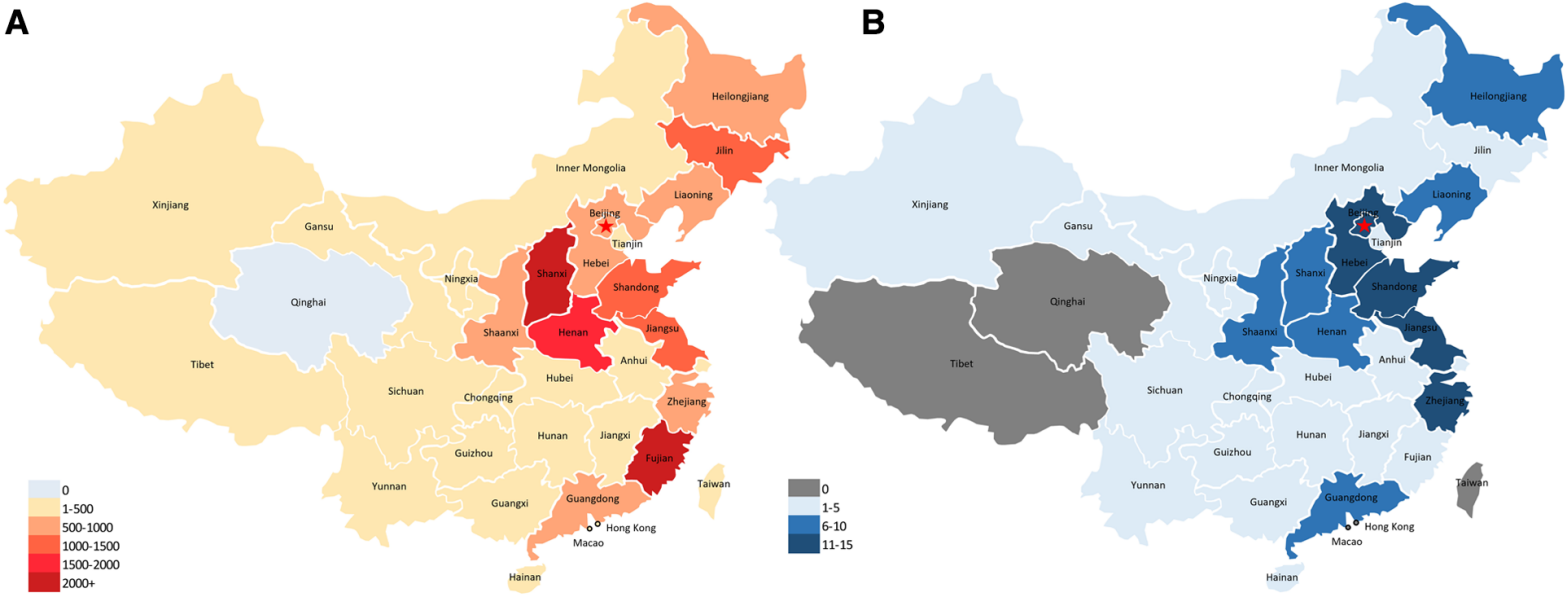
The geographical distribution of CR institutions and patients in the CACPR database in China, 2012–2021.

The annual number of registered institutions and patients is a significant indicator of the trends in this field. Nevertheless, these trends were unable to completely reflect the spatial distribution of CR proportions in China. Hence, sectional data for the corresponding regional distribution of CR were further compared, as shown in Figure 2.

Between 2012 and 2021, a total of 19,896 patients with CVD and 163 institutions from 33 provinces (except for Qinghai) were registered in the CACPR; of these, 9,810 (49.31%) were in the eastern region, 7,962 (40.02%) in the central region, 2,114 (10.63%) in the western region, and ten (0.05%) in Hong Kong, Macao, and Taiwan.

In terms of provinces, most patients were from Shanghai, Fujian, and Henan (more than 1,500). The proportion of patients referred for CR stratified by age, sex, complications, type of CR, risk stratification, and hospital type also showed a significant geographic difference (Table 1).

**Table 1:** Overall the distribution of CR population, institutions, and follow-up data across China, from 2012 to 2021.

		Number of Hospitals with CR	Number of registered patients with CR	Number of follow-up visits	Follow-up rate
Central	Shanxi (SX)	4	3,407 (17.12)	38 (1.5)	1.12%
	Jilin (JL)	4	1,090 (5.48)	7 (0.2)	0.64%
	Heilongjiang (HL)	7	985 (4.95)	167 (6.6)	16.95%
	Anhui(AH)	1	112 (0.56)	4 (0.1)	3.57%
	Jiangxi (JX)	2	185 (0.93)	51 (2)	27.57%
	Henan(HA)	9	1,868 (9.39)	156 (6.2)	8.35%
	Hubei (HB)	3	254 (1.28)	0	0.00%
	Hunan (HN)	3	61 (0.31)	12 (0.4)	19.67%
	Inner Mongolia (NM)	1	18 (0.09)	2 (0.1)	11.11%
Eastern	Beijing (BJ)	11	661 (3.32)	152 (6)	23.00%
	Tianjin (TJ)	4	171 (0.86)	100 (3.9)	58.48%
	Hebei (HE)	14	694 (3.49)	177 (7)	25.50%
	Liaoning (LN)	8	981 (4.93)	73 (2.9)	7.44%
	Zhejiang (ZJ)	15	590 (2.97)	153 (6.1)	25.93%
	Fujian (FJ)	2	3,258 (16.38)	57 (2.2)	1.75%
	Shandong (SD)	12	1,175 (5.91)	153 (6.1)	13.02%
	Guangdong (GD)	9	629 (3.16)	372 (14.8)	59.14%
	Hainan (HI)	3	89 (0.45)	9 (0.3)	10.11%
	Jiangsu (JS)	15	1,155 (5.81)	70 (2.7)	6.06%
	Shanghai (SH)	4	407 (2.05)	60 (2.3)	14.74%
	Guangxi (GX)	3	323 (1.62)	246 (9.8)	76.16%
Western	Xinjiang (XJ)	1	79 (0.4)	53 (2.1)	67.09%
	Chongqing (CQ)	2	493 (2.48)	59 (2.3)	11.97%
	Sichuan (SC)	7	213 (1.07)	11 (0.4)	5.16%
	Guizhou (GZ)	2	240 (1.21)	192 (7.6)	80.00%
	Yunnan (YN)	3	127 (0.64)	2 (0.1)	1.57%
	Tibet (XZ)	0	2 (0.01)	0	0.00%
	Shaanxi (SN)	9	587 (2.95)	123 (4.9)	20.95%
	Gansu (GS)	0	5 (0.03)	2 (0.1)	40.00%
	Qinghai (QH)	0	0	0	0
	Ningxia (NX)	1	27 (0.14)	0	0.00%
Hong Kong, Macao, and Taiwan	Hong Kong	0	7 (0.04)	1 (0.1)	14.29%
	Macao	0	1 (0.01)	0	0.00%
	Taiwan	0	2 (0.01)	1 (0.1)	50.00%
Total		159	19,896	2,503	100

### Descriptive analysis of CR population in the database

**Figure 3:**
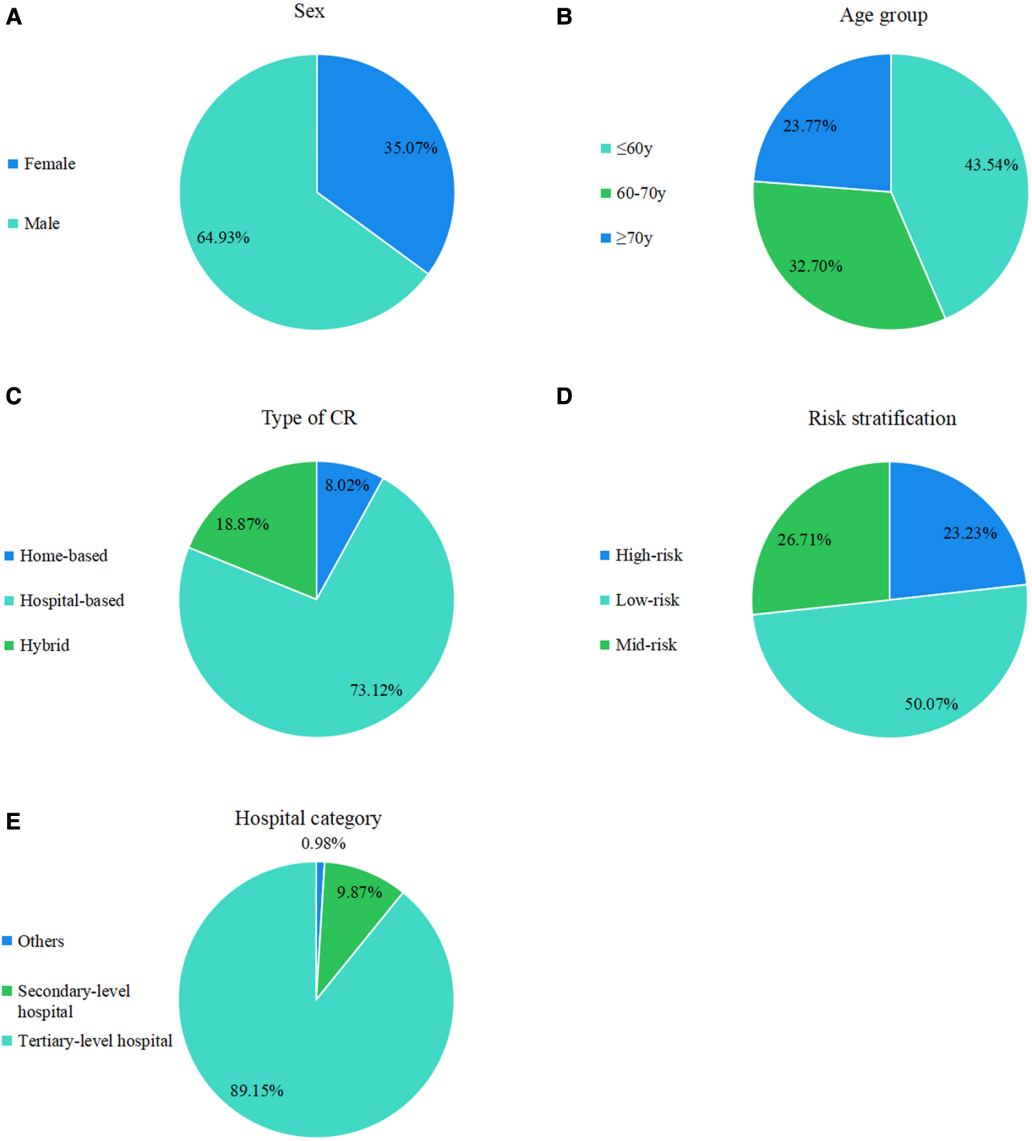
Registered CR distribution in the population grouped by sex, age, disease, risk of stratification, type of CR (home-based, hospital-based, and hybrid), and hospital category (tertiary-level, secondary-level, and others).

Data of 25,789 patients were obtained from the database between 2012 and 2021. Regarding empty data fields in this database, which were viewed as missing data, we finally extracted data of 19,896 patients (64.93% male vs. 35.07% female), of which 8,662 (43.54%) were younger than 60 years, 6,505 (32.70%) were between 61‒70 years, and 4,729 (23.77%) were ≥71 years. Among these participants, 73.12%, 8.02%, and 18.87% choose hospital-based, home-based, and hybrid CR, respectively. Only 23.23% were in the high-risk stratum, whereas 26.71% were in the mid-risk category, and 50.07% in the low-risk stratum. Patients registered for various types of CR model. The most common model was the hospital-based model (73.12%). Of the 159 CR institutions, 17,738 (89.15%) were tertiary-level hospitals (Figure 3).

The basic characteristics of the populations are summarized in Table 2.

**Table 2:** The characteristics of CR populations in the CACPR database, from 2012 to 2021.

Variables		Total	Distribution			
			Central	Eastern	Western	Hong Kong, Macao, and Taiwan
Gender	Male	12,919 (64.93%)	4,746 (59.61%)	6,713 (68.43%)	1,453 (68.73%)	7 (70.00%)
	Female	6,977 (35.07%)	3,216 (40.39%)	3,097 (31.57%)	661 (31.27%)	3 (30.00%)
Age						
Age group	≤60 y	8,662 (43.54%)	3,730 (46.85%)	4,077 (41.56%)	847 (40.07%)	8 (80.00%)
	61–70 y	6,505 (32.70%)	2,642 (33.18%)	3,186 (32.48%)	675 (31.93%)	2 (20.00%)
	≥71 y	4,729 (23.77%)	1,590 (19.97%)	2,547 (25.96%)	592 (28.00%)	0 (0.00%)
Risk stratification	Low-risk	9,961 (50.07%)	3,761 (47.24%)	5,318 (54.21%)	873 (41.30%)	9 (90.00%)
	Mid-risk	5,314 (26.71%)	2,496 (31.35%)	1,991 (20.30%)	826 (39.07%)	1 (10.00%)
	High-risk	4,621 (23.23%)	1,705 (21.41%)	2,501 (25.49%)	415 (19.63%)	0 (0.00%)
Type of CR	Home-based	1,595 (8.02%)	811 (10.19%)	647 (6.60%)	136 (6.43%)	1 (10.00%)
	Hospital-based	14,547 (73.12%)	6,093 (76.53%)	6,918 (70.52%)	1,527 (72.23%)	9 (90.00%)
	Hybrid	3,754 (18.87%)	1,058 (13.29%)	2,245 (22.88%)	451 (21.33%)	0 (0.00%)
Level of hospital	Tertiary-level	17,738 (89.15%)	6,779 (85.14%)	8,920 (90.93%)	2,029 (95.98%)	10 (100.00%)
	Secondary-level	1,964 (9.87%)	1,164 (14.62%)	721 (7.35%)	79 (3.74%)	0 (0.00%)
	Others	194 (0.98%)	19 (0.24%)	169 (1.72%)	6 (0.28%)	0 (0.00%)
Complications						
CHD		11,157 (56.08%)	5,235 (65.75%)	4,525 (46.13%)	1,395 (65.99%)	2 (20.00%)
Hypertension		9,136 (50.23%)	3,076 (44.77%)	4,894 (52.65%)	1,161 (57.70%)	5 (50.00%)
Hypercholesterolemia		4,295 (24.59%)	1,662 (25.68%)	2,115 (23.33%)	516 (26.90%)	2 (20.00%)
Diabetes		3,716 (20.85%)	1,081 (16.11%)	2,103 (22.98%)	531 (27.20%)	1 (10.00%)
OSAHS		209 (1.17%)	33 (0.49%)	124 (1.36%)	52 (2.63%)	0 (0.00%)
Angina		5,833 (32.57%)	2,355 (35.29%)	2,687 (29.13%)	790 (39.44%)	1 (10.00%)
post-MI		1,417 (7.85%)	406 (6.02%)	790 (8.52%)	220 (10.90%)	1 (10.00%)
post-PCI		3,231 (18.07%)	984 (14.89%)	1,797 (19.45%)	449 (22.18%)	1 (10.00%)
post-CABG		281 (1.55%)	87 (1.29%)	145 (1.56%)	49 (2.42%)	0 (0.00%)
MS		6,646 (42.85%)	2,421 (39.54%)	3,582 (47.45%)	638 (34.81%)	5 (83.33%)
EECP		2,973 (23.95%)	1,060 (21.02%)	1,627 (24.92%)	286 (34.13%)	0 (0.00%)
IPAQ	Lack of exercise	6,696 (49.71%)	2,813 (49.49%)	3,418 (51.97%)	459 (38.28%)	6 (60.00%)
	Up to the standard	6,316 (46.89%)	2,805 (49.35%)	3,074 (46.74%)	434 (36.20%)	3 (30.00%)
	Promoting-health exercise	458 (3.40%)	66 (1.16%)	85 (1.29%)	306 (25.52%)	1 (10.00%)
Follow-up		2,503 (12.58%)	435 (5.46%)	1,376 (14.03%)	690 (32.64%)	2 (20.00%)
Medicine						
Aspirin		12,874 (74.10%)	5,393 (81.56%)	6,063 (67.82%)	1,414 (78.08%)	4 (40.00%)
*β* blocker		9,468 (57.17%)	3,554 (58.60%)	4,922 (56.01%)	988 (58.22%)	4 (40.00%)
ADP antagonist		6,415 (40.57%)	2,400 (41.38%)	3,299 (39.32%)	712 (44.14%)	4 (40.00%)
Statin		13,934(81.27%)	5,380(84.43%)	6,988(78.18%)	1,561(85.53%)	5(50.00%)
ACEI/ARB		7,137(44.18%)	2,327(39.72%)	3,971(46.21%)	834(49.32%)	5(50.00%)

CHD, coronary heart disease; OSAHS, obstructive sleep apnea-hypopnea syndrome; MI, myocardial infarction; PCI, percutaneous coronary intervention; CABG, coronary artery bypass graft; MS, coronary artery bypass graft; EECP, enhanced external counterpulsation; IPAQ, International Physical Activity Questionnaire; ADP, adenosine diphosphate; ACEI/ARB, angiotensin-converting enzyme inhibitor/angiotensin receptor blocker.

**Figure 4:**
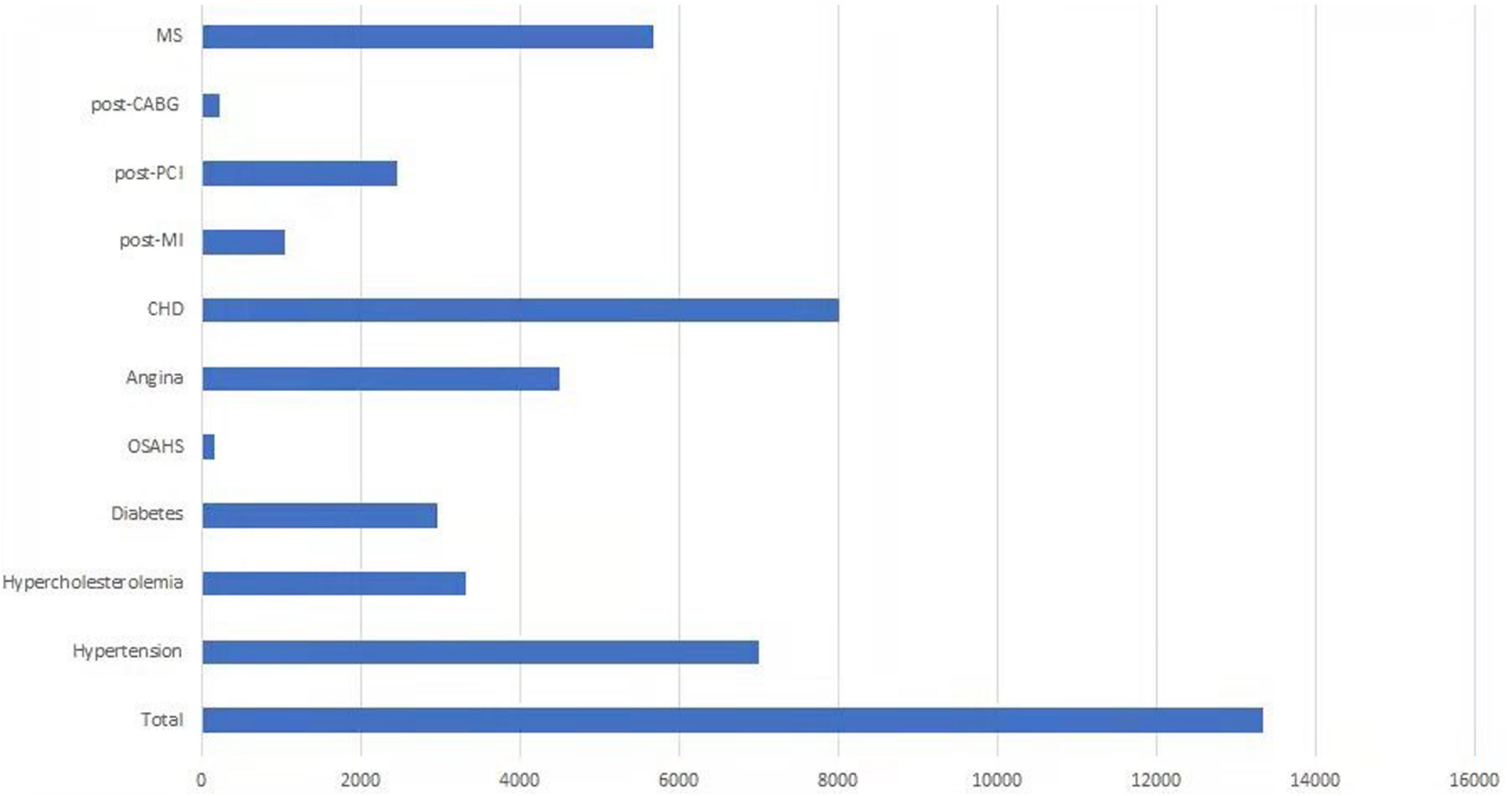
The diseases before CR across geographic regions registered in the database.

The three leading diseases before CR across geographic regions were CHD, hypertension, and metallic syndrome (MS) (Figure 4).

Among the 19,896 cases, only 1,817 (9.1%) completed the 36 sessions of CR and uploaded their CPET examinations.

### ANCOVA between different groups

Table 3 presents the results of the ANCOVA test, which revealed a significant between-group difference in exercise capacity adjusted to the baseline values of 6 MWT (*F* = 16.188, *P *= 0.000), peak VO_2_ (*F* = 4.96, *P *= 0.0007), peak Mets (*F* = 11.784, *P *= 0.000), and VO_2_-AT (*F* = 10.964, *P *= 0.000). From the results, the hybrid CR group showed significantly better outcomes than those in the home-based and hospital-based CR groups (*P *< .05).

**Table 3:** The results of the ANCOVA analysis.

Dependent variables	Baseline value			Post CR program			Post-treatment difference			
	Home-based CR^a^	Hospital-based CR^b^	Hybrid CR^c^	Home-based CR^a^	Hospital-based CR^b^	Hybrid CR^c^	F	*R* ^2^	*P* value	partial *η*^2^
6MWT	524.37 ± 147.18	463.02 ± 80.20	459.24 ± 111.05	547.95 ± 136.65	486.90 ± 91.23	510.97 ± 107.25	16.188	0.76	0.000	0.056
									(a, b^ns^, a, c*, b, c*)	
Peak VO_2_	18.93 ± 5.28	18.28 ± 5.01	17.09 ± 4.55	20.02 ± 5.28	19.6 ± 5.1	18.97 ± 5.15	4.96	0.698	0.0007	0.005
									(a, b^ns^, a, c*, b, c*)	
Peak Mets	5.55 ± 1.65	5.30 ± 1.49	4.88 ± 1.32	5.91 ± 1.63	5.73 ± 1.56	5.62 ± 1.52	11.784	0.603	0.000	0.013
									(a, b^ns^, a, c*, b, c*)	
VO_2_-AT	12.48 ± 3.84	12.53 ± 3.75	12.63 ± 3.58	13.35 ± 3.95	13.36 ± 3.72	14.04 ± 4.19	10.964	0.575	0.000	0.012
									(a, b^ns^, a, c*, b, c*)	

partial η^2^, effect size for ANCOVA; ns, non-significant.

* *p* < 0.05.

6MWT, 6-minute walk test; Peak VO_2_, peak oxygen uptake; VO_2_-AT, oxygen uptake at the anaerobic threshold.

^a^Home-based CR; ^b^Hospital-based CR; ^c^Hybrid CR.

## Discussion

Despite many efforts made to promote CR services, there were only 159 institutions registered in the database, which is less than that in other countries (12). Moreover, we observed that the number of institutions implementing CR increased from one in 2013 to 149 in 2021, and the overall number of patients that had undergone CR increased from 15 in 2013 to 5,183 in 2021, with differences in age, sex, and disease subgroups. The development of the social economy and the increased health awareness among residents may explain this phenomenon. However, the growth in CR remains insufficient to meet the actual needs of the Chinese CVD population.

To the best of our knowledge, this study is the first in China to report CR distribution and dissemination trends over time on a nationwide level. Our findings are fundamental to understanding the current state of CR and to help develop future strategies to improve CR delivery in China.

This study revealed that the participation rate of men in CR was higher than that of women, with a male-to-female ratio of 1.8:1, similar to that in the United States, and Canada (21, 22). Furthermore, consistent with previous reports, the larger participating group was more likely to be the younger group (≤60 years old) (23, 24). Lack of awareness, comorbidities, higher burden of frailty, fear of physical pain with exercise, and traffic difficulties are factors that contributed to this situation.

In China, healthcare providers reported that their hospitals’ lack of CR space, special equipment, and specific personnel training contributed to the underutilization of CR (17). According to size, bed, technology, equipment, and health insurance coverage, China’s hospitals are divided into primary, secondary, tertiary, and private hospitals. Compared with other hospitals, tertiary-level hospitals had the most advanced clinical skills and the largest number of patients and could supply high-quality health resources and services (25). For tertiary-level hospitals, health providers may be more informed of the benefits and necessities of CR than those at secondary-level hospitals.

Furthermore, the higher overall participation rate in tertiary-level hospitals than in other hospitals may be partially explained by the larger eligible patient mix at tertiary-level hospitals. Adequate physical space and equipment, and a professional CR team have also been identified as contributing factors to CR enrollment (26). Therefore, institutions with CR programs tend to be tertiary-level hospitals.

Although all regions showed a remarkable increase in the last decades; however, geographical disparities still existed within some provinces considered “CR deserts” such as Qinghai, which is an important factor for patients not participating in CR. Our report revealed that 159 institutions were registered in the database until 2021. Most CR patients and institutions are located in the eastern region of China, indicating that well-developed economies and regions with dense populations are more likely to undergo CR. As a developing country, China faces uneven distribution and inequalities in access to medical resources across regions (27, 28). Although the amount of medical resources has increased steadily in China, most are concentrated in the economically developed eastern region. Conversely, the central and western regions are economically backward, with a vast area and sparse population, and lack high-quality medical resources. The differences and unequal distribution in the geography of medical resources will inevitably lead to an imbalance in CR resources, with an impact on CR participation. In previous studies, the presence of CR centers in each region increased the possibility of patient participation and adherence (29, 30). Although CR resources are related to economic development, other factors, including the attention of the government or awareness of CR providers, may be more important, which would help explain why some eastern provinces were low-ranking, such as Beijing and Tianjin.

In our study, although most patients underwent hospital-based CR (73.12%), the hybrid model showed greater improvements in exercise capacity over the post-rehabilitation period compared to the hospital-based and home-based groups (*p* < 0.01). Pratesi et al. concluded that hybrid CR could improve functional exercise capacity and lower limb muscle strength compared to standard CR in patients aged >75 years, with similar benefits observed in our study (31). The benefit of this model is also evident in a few other studies (32, 33). During the coronavirus 2019 pandemic, people were encouraged to work and stay at home to keep up with physical distancing, and outpatient CR programs were postponed. A novel model named exercise-based hybrid CR is an effective strategy to improve exercise capacity and quality of life in patients with acute and chronic diseases (34). Compared to traditional CR programs, the hybrid model offers several potential advantages. It could facilitate eligible patients who are unable to visit rehabilitation centers for various reasons, decrease medical costs on CR, and improve patient satisfaction and adherence to the proposed CR. The approach is rather new and cost-effective, can even be generalized to more eligible CR patients, and needs to be widely developed both in China and worldwide.

From our findings, follow-up compliance after CR remains suboptimal, with only 12.58% of patients visiting for a survivorship visit. Further studies should focus on identifying factors associated with nonadherence to follow-up and apply effective strategies to improve follow-up visits after the CR program.

Identifying associated barriers is crucial to developing strategies to improve CR participation, which comprises both modifiable and non-modifiable factors, such as patient factors, healthcare providers, CR institutions, and geographic factors.

Our study suggests a challenge in China’s lack of available CR institutions, in which eligible patients could potentially participate. Although the availability of CR in China is low, a higher volume of cardiovascular surgery and its upward trend have been reported by the Committee of the Report on Cardiovascular Health and Diseases in China. According to data from 2017, 753,142 patients underwent cardiovascular interventional therapy, with the number growing steadily (35). It is noteworthy that in China, medical resources tend to be focused on surgery, but not on prevention and rehabilitation services such as CR. The Chinese government approved the plan named “Healthy China 2030,” which aims to improve national health and prevent disease in the coming years. There is a call for health policymakers to reset the priorities of healthcare resources and provide adequate preventative care. Moreover, it is necessary for healthcare professionals to underscore the importance of shifting from a traditional treatment model to a preventive-rehabilitation model. We hope that establishing and perfecting a practical preventive-treatment-rehabilitation system would help reduce disease mortality and improve the well-being of Chinese people in the future.

Different geographical locations, economic development levels, manpower, CR facilities, rehabilitation environments, and national policy support have all affected the development of regional CR.

The study had some limitations and caution is necessary when interpreting some findings of the study. First, CACPR does not include individual institutions in which CR programs remain unidentified. Many CR centers are included in the cardiology department, which is not a member of the committee. As CR develops in China, the committee allows many of the newer or smaller CR institutions to participate in the program, which is excluded from the database. Second, inter-region variation in what constitutes CR outcomes may be present; hence, bias should be considered. Third, the CACPR database lacks some information, such as patients’ attitudes toward CR, medical insurance, transport distance, and detailed information on CR institutions, including beds and equipment, which have been reported to be relevant to CR participation. Finally, the details of the CR procedure in each center remain unclear.

## Data Availability

The original contributions presented in the study are included in the article, further inquiries can be directed to the corresponding authors.
